# Intelligent Sensing Technologies for the Diagnosis, Monitoring and Therapy of Alzheimer’s Disease: A Systematic Review

**DOI:** 10.3390/s21124249

**Published:** 2021-06-21

**Authors:** Nazia Gillani, Tughrul Arslan

**Affiliations:** School of Engineering, University of Edinburgh, Edinburgh EH9 3FF, UK; Tughrul.Arslan@ed.ac.uk

**Keywords:** Alzheimer’s disease, intelligent sensors, smart sensors, smart devices, robots, smart homes, remote health monitoring, activity monitoring, user-centred design

## Abstract

Alzheimer’s disease is a lifelong progressive neurological disorder. It is associated with high disease management and caregiver costs. Intelligent sensing systems have the capability to provide context-aware adaptive feedback. These can assist Alzheimer’s patients with, continuous monitoring, functional support and timely therapeutic interventions for whom these are of paramount importance. This review aims to present a summary of such systems reported in the extant literature for the management of Alzheimer’s disease. Four databases were searched, and 253 English language articles were identified published between the years 2015 to 2020. Through a series of filtering mechanisms, 20 articles were found suitable to be included in this review. This study gives an overview of the depth and breadth of the efficacy as well as the limitations of these intelligent systems proposed for Alzheimer’s. Results indicate two broad categories of intelligent technologies, distributed systems and self-contained devices. Distributed systems base their outcomes mostly on long-term monitoring activity patterns of individuals whereas handheld devices give quick assessments through touch, vision and voice. The review concludes by discussing the potential of these intelligent technologies for clinical practice while highlighting future considerations for improvements in the design of these solutions for Alzheimer’s disease.

## 1. Introduction

Globally, neurological diseases (ND) are the primary cause of disability-adjusted life years (DALYs) [1]. Of these, stroke and Alzheimer’s along with other types of dementia are the two leading causes of DALYs [2]. Moreover, neurological diseases are the second leading cause of death all over the world [1]. People suffering from neurodegeneration undergo neuronal structure and functional losses. These abnormal structural changes in the brain provide a hindrance to normal brain function. As in the case of Alzheimer’s, the typical neuropathological hallmarks are the accumulation of beta-amyloid plaques and the formation of protein tau-tangles beside others [3]. These lesions provide interruptions in the synaptic transmissions from one neuron to another. The neurons start to lose their connections with the other neurons in the brain [4]. This results in the deterioration of the normal brain and human body functions [5]. Thus, the people that suffer from neurodegenerative diseases such as Alzheimer’s slowly start to lose their cognitive abilities and motor functions. This eventually renders them to be dependent on others for carrying out their activities of daily life (ADLs). To date, no standard cure has been put into practice for these conditions, although there have been extensive attempts in this regard [6].

Besides genetics and inheritance, age is one of the greatest risk factors for Alzheimer’s disease (AD). According to the 2020 annual report by the Alzheimer’s Association, 3% of people between the age of 65 and 74 years are currently suffering from Alzheimer’s disease. This number drastically increases with the increasing age window. This is evident from the statistics that show 17% of the people aged from 75 to 84 years, and 32% of the total people aged 85 and above are currently suffering from Alzheimer’s disease [7]. Neurodegeneration is relatively a slow process. However, once started, it cannot be reverted.

At present, the diagnosis of Alzheimer’s disease heavily depends on neuroimaging techniques. These tests include brain scans such as Magnetic Resonance Imaging (MRI), Positron Emission Tomography (PET) and Computed Tomography (CT). These scans although being effective, prove to be a challenge for people with neurological conditions. These methods are bulky, expensive and even invasive as in the case of PET scan. Dedicated lab requirements result in random episodic monitoring of the Alzheimer’s patient. This increases the chance for late detection of further neuronal damage. Moreover, the majority of the existing therapeutic solutions for Alzheimer’s include physical exercises to retain the physical functionality of the body and cognitive exercises for strengthening the existing brain cells. However, for these type of solutions, dedicated time and frequent or continuous help of either a clinician, nurse, formal or informal caregiver are required. This all amounts to the social, emotional, economical and physical burden on the caregivers. Given the progressive nature of the disease, the individuals suffering from it must be monitored regularly or continuously.

With the advancements in ubiquitous and pervasive computing, cloud and wireless technology, topping it all up with Artificial Intelligence (AI), the literature is rich with their application in dementia [8,9,10]. The proliferation of miniature sensors in smart devices such as smartphones and tablets, and ambient sensors that are designed for smart homes are playing a substantial part in the remote and continuous monitoring of Alzheimer’s disease [11]. Sensors form the backbone of these intelligent systems which enable these to sense various signals of interest from the environment. These sensors monitor signals from the humans and as well as the surroundings thus providing contextual information. These signals are then monitored and processed by embedded processors which provide an automatic response through actuators. This feature of the provision of apt feedback and response distinguishes these smart and intelligent technologies from the conventional ones [12,13,14]. This particular strength can be used for the management of diseases where continuous monitoring and adaptive responses are required, such as in Alzheimer’s disease. These technologies have the potential to provide autonomous care along with continuous yet remote monitoring of the patient improving their quality of life [15].

A handful of review studies exist that focus on the complex and specific needs of Alzheimer patients using assistive technology. Costanzo et al. [16] talk about the treatment and diagnostics of Alzheimer’s disease and mild cognitive impairment (MCI) with the help of telemedicine. They emphasize the fact that the use of technological services such as emails and video conferencing can prove effective sources for the detection and follow-up between the patient and the doctors. The work in [17] presents a scoping review of the general technological solutions for AD patients. Pillai and Bonner-Jackson [18] provide an overview in their study on the randomized clinical trials for AD patients using general information and communication systems. The systems mentioned in their study are used for monitoring cognitive and functional decline. The study outlines the strengths and weaknesses of these systems focusing on randomized clinical trials; however, it includes published articles until 1 September 2014. Hence, it is not an up-to-date overview of these solutions anymore. Elfaki and Alotaibi [19] present an overview of only the mobile health applications used for the assistance of AD. Studies [20,21] deal with the role of Virtual Reality Technology used for the diagnostics and cognition training of Alzheimer patients.

Based on the existing reviews, the following research gaps are identified:

- The existing reviews focus on the role of telemedicine or the general information and communication technologies for Alzheimer’s disease [16,17]. No recent review exists that focuses on the management of Alzheimer’s disease alone, using intelligent systems and devices.
- The existing review studies focus on a sub-class of intelligent or smart assistive technologies such as mobile phones or virtual reality for AD [19,20,21] and do not cover a broader spectrum of intelligent feedback systems.
- Some of the review studies are general in nature, covering intelligent technological solutions for all types of dementia, including Parkinson’s disease, etc., such as in [14]. This hampers the understanding of how these intelligent solutions can effectively be used for the complex and intricate needs of Alzheimer’s patients.
- The existing reviews either focus on part of the disease progression such as the early detection of AD [9,20,22] or therapy [21] or only on the monitoring of the functional decline of AD patients [18]. No review study exists that categorizes the intelligent solutions for the complete progression of Alzheimer’s disease, hence, providing a comprehensive picture of the role of these sensing technologies for Alzheimer’s disease.

Thus, there is a need for a recent up to date review that highlights the existing intelligent and smart technologies that are capable of assisting and managing Alzheimer’s disease. This review study aims to provide an up-to-date state of the knowledge for the intelligent assistive technological solutions for Alzheimer’s disease by searching the literature in the last five years. Moreover, according to [14], the number of intelligent assistive technologies is doubling every five years. Hence, the current systematic review intends to address the above-mentioned research gaps and contributes to the literature in the following ways:

- It provides a detailed overview of the recently researched intelligent technologies mentioned in the literature, focusing only on Alzheimer’s disease. Thus, the findings of the review will help in translating the researched solution into clinical practice as well as remote diagnosis and monitoring of the disease. The review tends to raise awareness of the potential of these automated intelligent solutions among the stakeholders (clinicians, caregivers and Alzheimer’s patients). Moreover, emphasizing only on Alzheimer’s disease will facilitate future research focus on the complex needs and particular symptoms of Alzheimer’s patients.
- The review covers a broad spectrum of intelligent solutions designed and researched for the complete progression (diagnosis, monitoring and therapy) of Alzheimer’s disease. This will aid in provision of a broader picture and a comprehensive understanding of the role of these sensing technologies throughout the Alzheimer’s progression.
- The review study also aims to evaluate the efficacy and usability of these proposed solutions. For this purpose, the review focuses on the studies, identified in the searched databases, which implemented the solution and involved Alzheimer patients for the validity of their proposed intelligent solution.
- The review study discusses the practical implications of these intelligent solutions. Moreover, it also highlights the limitations of the current researched studies and the areas that need consideration for better and improved solutions, thus indicating new directions for researchers in this field.

The remaining review article is organized as follows: Section 2 describes the materials and research methodology undertaken for the systematic review. It states the research questions for the current study, followed by the detailed description of the database search and the articles’ screening methodology. Section 3 gives a comprehensive analysis of the results identified in the literature search. This is followed by Section 4, which discusses the results, the social and economic impact of these technologies and how they can play a vital role in improving the quality of life of Alzheimer patients and of those who are responsible for caring for and treating them. This section also highlights the current limitations identified in the searched studies and provides suggestions for future improvements. Finally, Section 5 provides the conclusion of the review study.

## 2. Materials and Research Methodology

To provide an oriented and impactful outcome, the research questions addressed in our review article are explicitly stated in Table 1. The first two questions highlight the overall motivation behind the study. They focus on the kind of intelligent sensing technologies that exist in the literature for the care of Alzheimer’s disease and the purpose served by these technologies, in its management. Keeping in view the scope of the current article, we tend to focus on intelligent sensing technologies that can provide low-cost solutions and do not require dedicated labs or expensive equipment for the diagnosis, treatment and monitoring of Alzheimer’s disease patients. The third research question is a focused research question (FRQ). It tends to focus on how the different stakeholders are involved in the design of these systems and devices and what are the challenges faced and limitations in the existing solutions. Emphasis is given on how the proposed designs or prototypes have used the user-centric design approach.

### Systematic Search Methodology

A systematic search was carried out using the PRISMA guidelines [23]. Four databases were explored. The database search engines accessed were: Scopus, PubMed (Medline), Web of Science and IEEE Xplore. The articles searched were from 2015 until the 6 January 2021, the date on which the databases were searched. Only English language articles indexed in the above search engines were considered for inclusion in this review. The document type selected for the current review included only ‘Articles’ that were published in the journals whereas all remaining document type categories, such as books, book chapters, conference proceedings, etc., were excluded.

Significant consideration was given while choosing the keywords for searching the required studies. Various combinations were tried. As an example, the very first combination that was used by the authors was “Alzheimer” AND “smart OR intelligent” AND “sensor OR device”. After seeing the results, a modification was made by adding the keywords ‘assist, assistive or robot’ with ‘device OR sensor’, to widen the spectrum of the required results. Hence, the final combination of the keywords used was the combination of: (Alzheimer) AND (smart OR intelligent) AND (device OR sensor OR assist OR assistive OR robot). These keywords were searched in the data fields of the ‘Article title, Abstract and keywords’ in the advanced search option. For example, the search strategy in the IEEE Xplore database performed on the 6 January, 2021 was: (“All Metadata”: Alzheimer) AND (“All Metadata”: smart OR “All Metadata”: intelligent) AND (“All Metadata”: device OR “All Metadata”: sensor OR “All Metadata”: assist OR “All Metadata”: assistive OR “All Metadata”: robot). The same was repeated for Scopus, Web of Science and PubMed databases.

This search methodology resulted in a total of 253 journal articles (Scopus: 63, Web of Science: 60, PubMed: 23 and IEEE Xplore: 107). Next, 57 duplicates were removed, first by using the EndNote software and then by screening the articles for the title, abstract and author names. Based on the scope and inclusion criteria, 121 articles were removed after screening the title and abstracts. Finally, reading of the full text of the remaining articles was carried out by the first author and thorough discussions between both the authors led to the inclusion of 20 articles. The inclusion and exclusion criteria used by the authors is described in detail below.

To be included in the current review study the articles needed to

- Have included an intelligent or smart solution for the diagnosis, monitoring, assistance or therapy of Alzheimer’s disease.
- Include Alzheimer’s or MCI patients along with sufficient information about the methodology of the design and study. However, those studies were also included whose participants were older adults aged 55 years and above. The studies including young healthy controls were also included if they were used as a control group, acting as comparators to the AD patients.
- Have sufficient information about the evaluation metric and result validation.
- Be written in the English language.
- Be a published journal article.
- Be published between the years 2015 to 2020.

However, the articles were not included if

- They had AI algorithms implemented on neuroimaging scans, EEG signals or similar data from existing Electronic Medical Records.
- Used specialized or dedicated lab equipment for the detection of metal ions, chemical structures or drug delivery inside the body.
- Were theoretical such as interviews or perspectives from caregivers.
- Implemented activity recognition or systems to mimic human behaviour for smart future agents.

**Figure 1:**
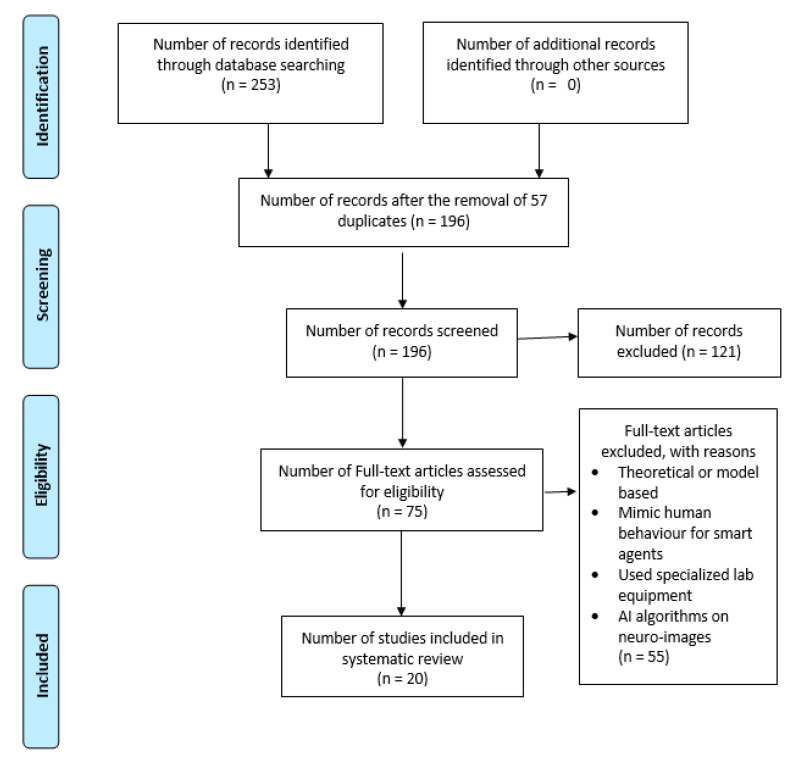
PRISMA flow diagram for search methodology.

Figure 1, below, describes the PRISMA flow diagram for the searching, screening, eligibility and inclusion of the identified articles for the current review.

## 3. Results

Following the research methodology detailed in Section 2, a total of 20 articles were included in the analysis and classification for the current review paper. Two broad categorizations of the intelligent technologies used in the article studies were observed. These include self-contained handheld devices and the distributed systems. Out of the studies selected, 11 fell under the category of distributed systems. These distributed systems included the installation of various sensors in a residential setting or a care home. These sensing nodes collected various signals from the patients and environment, sending them to the cloud or other processing units. After computations, suitable actuators in the homes were activated providing intelligent feedback to Alzheimer’s patients. The remaining nine studies dealt with the implementation of solutions involving technologies such as smart devices, the development of software applications that run on these smart devices, and virtual assistants such as robots.

The following subsections describe in detail the kind of sensing technologies and their supporting systems used in the proposed solutions. This addresses Research Question 1 which states that ‘What intelligent technological solutions exist in the literature that may help in the assistance and management (diagnosis, monitoring and therapy) of Alzheimer’s disease?’ The different study outcomes, that is, the various stages of disease profiling (diagnosis, monitoring and therapy), require different methodology and require the monitoring of different signals. Hence, the initial part of the results provides a description of the methodology, participants involved and the intervention used, describing the effectiveness and acceptability of the proposed solutions. This addresses Research Question 2 which states that ‘How do the proposed intelligent technological solutions help in the assistance and management (diagnosis, monitoring and therapy) of Alzheimer’s disease?’

To evaluate the effectiveness of the researched intelligent solutions, the comparator used in the study for result validation is also clearly reported. Some studies compared their results to a standard clinical test while others used a control group to explain their evidence-based outcomes. These descriptions then are followed by tables that further classify the studies implementing the distributed and self-contained solutions for Alzheimer’s disease. The tables summarize the duration, participants, technology used, study design and the particular measurements monitored by the sensors and devices for the study outcomes (diagnosis, monitoring and therapy). The tables also highlight the key findings, limitations and report whether the design or study included the user-centred design approach or not. These thus address the last Focused Research Question which states that ‘How are these intelligent systems and devices incorporating the user-centric design and what are the limitations in the existing designs implementations?’ The limitations and challenges are also further discussed in detail in Section 4 of the review article.

### 3.1. Distributed Systems

Distributed systems are systems that have multiple sensing nodes, data processing sub-systems and actuators that are activated according to the requirement and context as sensed by the sensors. These multiple components interact with each other through computer networks via network protocols. According to [6], the subsystems “host processes and use a set of protocols to aid in the coherent execution of the distributed activities”. The studies that realized these distributed systems were conducted longitudinally in either the care homes, nursing homes, labs or residential homes where the Alzheimer’s patients resided. The following subsections provide a classification of the studies based on their outcomes.

#### 3.1.1. Diagnosis of Alzheimer’s Disease

In the clinic, there are standard tests used for the diagnosis of Alzheimer’s disease. Besides the neuroimaging scans which indicate structural changes in the brain, there are other questionnaire-based tests as well. These tests evaluate the cognition of an individual through questionnaires or similar screening tools. Common examples include Clinical Dementia Rating (CDR) [24], Mini-Mental State Exam (MMSE) [25] and Montreal Cognitive Assessment (MoCA) [26]. However, these tests provide a sporadic and subjective assessment. The continuous monitoring of the vulnerable population can result in an early diagnosis as well as objective measurement for the detection of Alzheimer’s disease progression.

Autonomous detection of early-stage Alzheimer’s, MCI, was carried out in a study by Akl et al. in 2015 [27]. The study included 97 participants all aged 70 years or above with CDR score < 0.5 and MMSE > 24 at the start of the study. Each participant was living independently in a home equipped with smart sensors. They were continuously monitored for a period of 3 years. CDR and MMSE assessment was repeated by the end of each year, for the three years. For comparison, at the start of the study, participants were categorized as cognitively intact or baseline MCI. The data collected was that of walking speed and activity throughout the day. Passive infrared sensors, installed in the homes, were used to collect the walking speed data. Signal processing and machine learning algorithms (Support Vector Machine (SVM) and Random Forest (RF)) were applied to the data. The authors concluded that the most important measurements in detecting MCI were weekly trajectories, the variation coefficient of walking speed, coefficient of variation of the subject’s morning and evening walking speed along with their age and gender. In their study, they successfully distinguished MCI individuals from the cognitively healthy elderly.

In another study by Akl et al. in 2017 [28], the authors used the data from smart homes of 68 elderly patients having the same characteristics and in the same environment as mentioned in their earlier study [27]. However, in [28], they built generalized linear models (GLMs) of the everyday activities of the individuals and did not use any predefined measures for the detection of cognition. They concluded that by using normalized Kullback-Leibler divergence on the GLMs with 12-week window size, their technique resulted in a successful generalized solution for the detection of MCI. They also noticed that while the participants transitioned to the MCI stage there was no significant slowing in the gait speed.

A detection system based on Alzheimer disease ontology using agent-based technology, smart IoT devices including mobile phones and RFIDs was implemented by Kaur et al. [29]. Data collected from 374 patients with ages 60 to 96 years was used in the study. A cloud platform for the storage of the data and their processing was used. The detailed information of the patients including all the demographic, health (including symptoms) and MMSE data was fed to the smart system for the detection of Alzheimer’s disease. The study was qualitatively validated based on accuracy, usability, readability and convenience as compared to the similar four existing ontologies in the literature. Quantitatively, it was verified by using 100 patients out of the 374 for testing. Various classifiers were used such as Naïve Bayesian, J48 and the ontology Bayesian Network, with the latter performing the best. The measure used for assessing the accuracy of the system was the area under the curve (AUC) [30] with a value of 0.76. This was the greatest as compared to other classifiers used in the study. In addition to this Bayesian Network decision model, the system also monitored the physical location of the 100 patients using RFIDs that continuously updated the clinician through an android application developed for the purpose. The application also gave reminders to the patients for medicine and had memory recall exercises. Thus, assisting the patients as well as diagnosis.

In the study done by Alberdi et al. [31], the authors attempt to predict various symptoms that are an indication of Alzheimer’s disease. Smart-home sensor data were used to detect decline of cognition, changes in mobility and mood decline. The duration of the study was more than 2 years, and a total of 29 older adults were involved in the study. Various standard tests for mobility, cognition and mood were carried out at the beginning of the study and repeated after a period of every six months. For mobility, the tests included Arm Curl [32], Timed Up and Go Test (TUG) [33]. For cognitive decline, the standard test used was Repeatable Battery for the Assessment of Neuropsychological Status (RBANS) [34] and Prospective and Retrospective Memory Slips (PMRQ) while the mood was assessed using Geriatric Depression Scale (GDS) [35]. Based on the tests scores, the 29 participants aged 73–97 years old were classified as cognitively healthy (N = 13), at-risk (N = 10) and participants with cognitive difficulties (N = 6). Regression analysis was performed, and the study concluded that mobility, depression and cognition can reliably be predicted using smart home sensors. The results also indicated that mobility scores were correlated most with behaviour followed by cognition. The daily routine and socializing patterns of the participants were important indicators for the various symptoms’ detection. Moreover, the overnight activities such as visits to the toilet and sleep patterns and mobility routines were correlated with cognition. Finally, in-home and outing activity was considered to be related to a diagnosis of depression although it lacked statistical evidence.

#### 3.1.2. Continuous Monitoring of Alzheimer’s Patients

Alzheimer patients suffer a range of functional deterioration along with a decline in cognition. The continuous context-aware monitoring can help in timely detection of this descent in daily capabilities providing opportunities for timely interventions. The sensors used in the distributed systems can measure certain signals that may reveal information about the individual’s changing health and personal behaviour. Algorithms and models then can make a viable assessment to predict the declining functionality of the individual. The continuous monitoring of the individuals also enables them to assist either automatically using intelligent voice assistants, pre-recorded messages or can alert the caregiver for a timely intervention. The studies included in the review provide with assistance as described below:

(a) Detection of Abnormal Behaviour

Abnormal behaviour is one of the main challenges when it comes to Alzheimer’s patients. Alzheimer patients often suffer from agitation and decline in mood. Alvarez et al. [36] used a range of sensors including wearable bracelet, zenith camera, Microsoft Kinect v2, magnetic binary sensors and wireless sensor network beacons to detect abnormal behaviour. These were used in an indoor multi-patient setting. The signals measured from the sensors included physiological data such as heart rate and skin temperature along with motion and location tracking data. The study included 18 patients in a hospital setting, aged 55–94 years, however, not suffering from cognitive issues. These patients were classified as very ill, moderate ill and mildly ill by their doctors. The duration of the study was 10 weeks. A weighted probabilistic model was used depending on the fusion scheme of the collected data from the multiple sensors used. The system was able to detect abnormal behaviour with an accuracy of 98.4%. This decision was based on the correlation calculation of the activities performed by the individuals and where those particular activities were performed.

(b) Detection of Autonomy in Activity Performance

Automatic and objective detection of autonomy in the everyday tasks of Alzheimer patients was carried out by Karakostas et al. [37]. In total 109 participants were part of the lab-based study across two different geographical regions (France and Greece). From France, there was a total of 11 participants which included both, MCI patients and Healthy Controls (HC). Similarly, the remaining 98 participants were categorized as 27 AD, 38 MCI and 33 Healthy Controls (HC). These were assessed on MMSE scores (both sites), Functional Rating Scale for Dementia (FRSSD) and Functional Cognitive Assessment. The labs were instrumented with the necessary information computed technologies; video cameras, tags on house objects and mobile phones to run applications were used. The data collected from these sensors were used for the automatic detection of the (i) participants and (ii) tasks performed, and (iii) autonomy level was done using AI and signal processing algorithms on video signals. The instrumental Activities of Daily Living (iADLs) used for assessment were making an online payment, preparing a drink, medicine box preparation and talking on the phone. Results indicated a statistical correlation between the findings from both geographical regions. Moreover, using statistical analysis using the ANOVA (analysis of variance) test, the results confirmed that the autonomy level of HC is much better than MCI patients, and similarly, the autonomy level of MCI patients is better than that of AD patients.

(c) Provision of Assistance

Barley et al. [38] used a smart auto-prompting system to assist people with cognitive problems. The system developed was used for guiding the patients with cognitive problems or memory problems while they perform their iADLs. The eight activities performed included common household chores such as cleaning the kitchen, using the telephone, doing laundry, and cooking. The participants in the study included 15 elderly aged 59–91 years old. All of them met the criteria for mental disorders defined by the Diagnostic and Statistical Manual of Mental Disorders-IV-TR. A university furnished apartment fitted with smart sensors, actuators, speakers, cameras and TV screens was used as a testbed. The prompting system consisted of pre-recorded automated messages. If the user could not follow the instructions given by the auto-prompt system, they were told to go to the nearest TV screen. There a video with all the required steps for the particular task was shown on how to complete the activity they were having difficulty performing. Two experimenters observed the reactions of the elderly patients to how they responded to the automatic prompting systems. Qualitative assessment was carried out with a team of 4 experts from gerontology, nursing neuropsychology and assistive technology. It was concluded that context and the level of cognition level affects the acceptability of auto-prompting systems, as the people suffering from cognition problems showed levels of stress while responding to non-human prompts.

Similarly, in a study by Cavallo et al. [39], a system using smart domiciliary sensors was developed to assist Alzheimer patients and their caregivers. The design phase of the system involved a team of physicians, patients, caregivers and psychologists. Initially, they tried to assess specific user needs through interviews, hence, giving a good example of user-centric design. Based on the results, they identified four key concerns, including functional monitoring, alerting of an emergency, social behaviour and support for cognition. Based on these results, they designed solutions for the four domains mentioned. A total of 14 Alzheimer disease patients were recruited for the study, aged 80 years and above. According to MMSE, they were classified as AD-phase 1 and 2 patients. To address the concerns the researchers developed an in-home bed-chair monitoring system, patient location indicator and a home enter exit system. The sensors used for the bed-chair system were two metallic plates that acted as a closed switch when someone sat on them. The door open-close was monitored by a magnetic switch while to indicate the location the patients had to wear a device integrated with Global Positioning System (GPS) and Global System for Mobile Communication (GSM). All the sensors communicate with a remote computer monitored either by a caregiver or spouse of the patient through Zigbee. To support cognition, the system included the use of music and videos related to the past of the users for therapy purposes acting as stimuli. The duration of the study was not fixed, and only those systems were implemented in the home of the patients as per need or specified by the users themselves. The authors concluded that the acceptability and usefulness value of the implemented systems was 50% and 30%, respectively, as reported by the users after the study. Another important conclusion was that the wearable device was not suitable for AD patients with adverse symptoms.

Night-time wanderings are another common characteristic of Alzheimer’s patients. Radziszewski et al. [40] devised a system that tries to detect a night-time wandering of patients and tries to return them to their bed. The study included a single participant, a female aged 78 years old. According to her score on the MoCA test, she was an MCI patient. The system consists of 30 ambient smart-home sensors that were installed in her home where she lived alone. Moreover, a smart wristband was used as a wearable and smart home objects such as LED bulbs and lamps were also installed in the home. The first week of the study was used to test the sensors and understand the participant’s routine. The next two weeks were used for the testing phase of the study. In this phase, only the data were collected, but no assistance was provided. However, the caregiver was also involved at this stage for collaboration. K-means algorithm and clustering techniques were used to classify the patients’ day and night activities. Then actuators, speakers for voice messages and calming music apparatus were installed in the home for assistance, and after testing the system for a week, a 15-day validation study period was carried out. At the end of this, MoCA and Dementia Rating Scale (DRS) were performed again. The results indicated that the data collected in the 42-day study were insufficient for determining nocturnal patterns; however, the small clustering of activities gave an overall view to the caregiver for better assistance.

The system developed by Kaur et al. [29] also assists by generating automatic alarms and message reminders to help Alzheimer patients in memory. It also has cognitive support exercises, and through a GPS, it can guide an AD patient back to a rehabilitation centre.

(d) Monitoring Functional and Cognitive decline

In an 8-year longitudinal study carried out by Lyon and co-authors. [41], smart sensors were strategically placed in 480 homes of an elderly population. All the participants were 70 years or older in age and had scores MMSE > 24 and CDR ≤ 0.5 at the beginning of the study. The sensors include passive and wrist-worn sensors such as actigraph acting as ground truth. The sensors were used to monitor the gait, activities performed, mobility patterns, leisure time (computer usage and socializing) and medicine adherence of the residents of the homes. Statistical analysis, logistic regression along other predictive analysis were applied to the multi-domain data. Results showed that using multimodal data collected from sensors, they could identify decline in cognition, loneliness and mood anomaly within a short period. These were identified using the changes in mobility, socializing and activity patterns as observed by the sensors. Some of the symptoms of decline and Alzheimer disease progression were detected within six months. It is also noted that various behaviours such as speed of computer usage and sleep patterns are different for MCI patients as compared to people in the later stage of Alzheimer’s.

#### 3.1.3. Therapy and Rehabilitation

The effective use of assistive technology on the cognition, behaviour and functional challenges experienced by Alzheimer patients was evaluated by Lazarou et al. [42]. In their study, they recruited 18 patients (12 MCI, 6 AD patients) and divided them into 3 control groups. Each group had 4 MCI patients and 2 AD. The MCI patients were selected on Peterson criteria [43], and the AD participants were selected according to the Diagnostic and Statistical Manual of Mental Disorders (DSM-V) criteria [44]. The duration of the study ranged from 4 to 12 months. The interventions used were off-the-shelf IoT devices including wireless tags for in-home objects, Infrared (IR) motion sensors, sleep monitoring sensors, wearable band and ambient depth cameras. Sleep, motion and activity patterns were monitored for the first and second control group. Based on the information generated by these sensors, the first group received automatic, clinical updates, recommendations and alerts for their care and other non-pharmacological purposes. The second group received these automated prompts based on self-reported methods. The third group did not have the system installed in their homes neither did they receive any proposed interventions. All the participants underwent MMSE and various neuropsychological tests before and after the study. The study concluded that the group that was continuously monitored and received all the appropriate automatic, system-generated interventions, and hence, the timely treatment showed significant improvement in cognition by the end of the study. The cognitive and memory abilities of the patients in group 1 remained better intact, even better, as compared to group 2, which was receiving partial on-demand interventions. Moreover, the results of group 2 were better than that of the third group that received no treatment and no automatic monitoring system. Hence, the functional and cognitive decline of the third group progressed as expected in Alzheimer’s disease.

Table 2 provides a summary of all the studies implementing the distributed systems for the diagnosis, assistance and therapy of Alzheimer’s disease.

### 3.2. Handheld Devices

This section deals with the intelligent sensing technologies that are not spread throughout the care homes, nursing homes or individual’s smart home. These instead are based on interactive devices such as a smartphone or a tablet which takes the user input through touch, video or voice. Robotic interfaces, socially assistive robots and virtual assistants are also part of this category of intelligent sensing technologies. Mobile phones and tablets have software applications running on them that provide adaptive feedback to the user using the touch screens. Out of the 20 studies, 9 were categorized as those whose solutions involved self-contained devices. These devices were further classified into the following groups: smartphones/tablets, interactive robots, applications implemented on smartphones/tablets and smart furniture.

These devices are being used for the diagnosis, therapy and assistance of Alzheimer’s disease. These sensing technologies are playing their part in the diagnosis and detection of various Alzheimer related symptoms such as cognitive and memory decline, tremors and visio-motor coordination skills. For therapy purposes, there exist devices that are working to aid for medicine adherence of the Alzheimer’s patient. Moreover, devices and applications are implemented for cognition and memory exercises. Music therapy for mood-boosting purposes and for strengthening episodic memory is also being carried out. The following subsections provide a classification of the studies based on their outcomes.

#### 3.2.1. Diagnosis of Alzheimer’s Disease

Gonzalez et al. [45] designed a system that evaluates the memory of an individual. Their system includes a three-door cupboard installed with magnetic switch sensors for each door of the cupboard, a Raspberry Pi and an external battery. The 23 participants involved in the study include adults in the range of 18–60 years old. The young adults were included as a control group for comparison purposes. The attributes assessed in the study were memory and the reaction time of an individual. To validate the memory assessment, a shorter version of the standard test ‘face-name pairs’ [46] along with a self-reported memory test [47] were used as an evaluation and validation metric. The algorithm on the Raspberry Pi recorded information of the number of attempts of finding an object in the cupboard versus actually finding the object. An experimenter also independently observed the individuals during the task. The memory measurements from the study were compared with that of the standard tests. Pearson correlation test was performed. A high correlation between the results from the designed system and the memory tests were observed. Moreover, the study results indicate that no correlation exists between the reaction time of older adults versus younger adults.

The study carried out by Suzumura et al. [48] also tends to diagnose the decline in cognitive function using finger dexterity. They were able to do so using a smart terminal device ‘JustTouch’ which was used to assess the various finger tapping patterns. These tests included rhythmic (left or right hand and then both simultaneously) and sounded rhythmic (using either hand) patterns. The response time was also calculated with precision. The participants in the study consisted of 31 AD patients (mean age 74.2 years) and 15 people diagnosed with MCI (mean age 74.3 years). Healthy controls (HC) (mean age 74.6 years) also participated in the study. MMSE test was carried out for all participants at the start of the study. The statistical analysis using Spearman’s correlation coefficient shows that significant differences exist between the three groups (AD, MCI and HC) in the contact duration, rhythm and response time (lag time). A negative correlation was also indicated between the MMSE and contact duration of the touch screen.

Lee et al. [49] built a small play apparatus consisting of four different colours Light Emitting Diodes (LEDs), an Arduino board and a small speaker. This system is connected to a smartphone using Bluetooth. The system is capable of diagnosing cognition problems as well as providing treatment for them. The diagnosis is carried out by calculating the reaction rate as a response to the game. The cognition therapy is carried out by providing repeated cognition stimulations and taking care of the hand, vision and hearing coordination. The participants included in the study were healthy control adults in their 20 s and 70 s, and 10 measurements from each patient were taken while the other group consisted of 14 people suffering from cognition problems and MCI patients, and they were tested twice as much as those of the first group. It was noticed that the response rate for cognition of the elderly in their 70 s was 300 ms slower than that of those in their 20 s. However, the response time of the patients with cognitive issues was 9.64 s slower as compared to healthy controls in their 20 s while 5.53 times slower than those in their 70 s. Hence, the device was successful in differentiating the age-related as well as detecting impairment in cognition of the individuals.

Low-cost haptic feedback robotic interface was developed by Bartoli et al. [50]. The apparatus consists of a computer screen on which a visual target appears and a haptic interface. The apparatus was developed to detect the Visio-motor deficits of Alzheimer patients. The commercially available haptic device, Omni SensAble, was integrated with the Windows XP monitor using customized software. The device helps to draw the hand trajectory while the user tries to follow the visual target on the screen. The study consisted of controlled randomized trials. The control group involved 20 healthy, cognitively sound adults aged between 53–90 years, while the experimental group consisted of 20 AD patients aged 57–82 years of age. The reaction times, position tracking of the object and stability of the position tracking were observed for trials that lasted less than an hour. In addition, MMSE and standard vision tests such as Rey’s Figure copy-REY-Clock Drawing test were performed. Results of the study indicate that there was a marked correlation between cognitive abilities and memory to that of visio-motor performance. The experimental group was significantly slower in response time. Moreover, the Attentive Matrices test and MMSE were negatively correlated with reaction time. The tracking error also increased for the AD patients, and hence, the performance of position tracking was better with the cognitively well group.

#### 3.2.2. Assistance in Everyday Activities

Fardoun et al. [51] built a prototype system consisting of a smartwatch and mobile that helps Alzheimer patients in recognizing people. They made use of a smartwatch that had a built-in camera and cloud technology. The Alzheimer patient takes the picture of the person in front of him/her using the smartwatch. The picture is then sent to the mobile which is then compared to a pre-existing customized database on the cloud data. A face recognition algorithm recognizes the face and displays the information of the person on the screen of the smartwatch. This aids the Alzheimer’s patient to recognize the person, thus helping in memory rehabilitation. The prototype was tested on 41 people aged 55 to 72 years old that experienced no visual difficulties. Results show that although the system works well in face recognition, the vision of the information displayed on the small smartwatch screen proved to be challenging for the elderly especially those with Alzheimer’s.

Rudzicz et al. [52] made use of a human interactive robot named ED that could have a conversation and direct patients with cognition problems in their daily tasks. They assessed the feasibility of using such a robot in a nursing home-like setting, interacting with 10 AD patients simultaneously. The robot assisted the AD patients with verbal prompts after visually monitoring. The robot was movable and had a video display screen, speakers and cameras installed on it. Speech recognition was an integral part of the robot used in the study. A total of 10 AD patients along with their caregivers were recruited for the study. Throughout the study, the robot was teleoperated, and when a caregiver was required or intervened, the robot was no longer used for assistance in that particular task or time. It was used while guiding the patients to perform simple tasks that had a specific number of steps involved such as making tea. It also guided them or followed them to the washroom. It was noted that the patients seldom interacted with the robot in two way-conversations. It was better used when engaging in familiar tasks; however, human-human (patient-caregiver) interactions were more used by the patients in interview type conversations. The AD patients ignored the robot prompts over 40% of the behaviours that indicated confusion. However, the automatic speech recognition capability of the robot was assessed to be 70% which was greater than the already existing in the literature.

McGoldrick et al. [53] evaluated an application ‘MindMate’ that was developed to help and support people with later stages of Alzheimer’s (dementia). It was developed to assist in memory tasks by using reminders. The study was carried out to assess the usability of the application to aid in memory management. Three participants diagnosed with Alzheimer’s (at MCI stage) according to ICD-10 criteria (International Statistical Classification of Diseases and Related Health Problems) and reported to experience memory problems were recruited for the study. Moreover, partners (responsible for taking care of the patients) also participated in the study. Baseline performance was recorded by the carers of the patients for the first phase without using the application for five to seven weeks. Then, a weekly assessment was made for 5 weeks after the intervention was introduced. One of the AD patients withdrew from the study in the first week of the intervention. The patient was irritated with the reminders and the difficulty of turning them off. In addition to observational data analysis, Tau-U (Unified Theory of Acceptance and Use of Technology) [54] was also carried out. Results showed significant improvement in the memory performance for the two remaining patients.

#### 3.2.3. Therapy and Rehabilitation

Lyu et al. [55] developed a system consisting of a smart robotic dog along with an adaptive fuzzy logic inference module and cloud technology. A corresponding web application along with a few sensors such as pulse oximeter, electroencephalogram (EEG) and smartwatch was also part of the system. The overall system was used to detect symptoms of pneumonia in AD patients. The robotic dog was used to detect the emotions of the AD patient and respond to them accordingly. When simulated, it played music. For AD patients, music therapy is used to calm their mood and sometimes it is used for their memory rehabilitation. Furthermore, depending on the beat of the music, it can also encourage movements in AD patients [56]. The smart dog provided the patients with companionship as well as it was designed to lessen the caregiver burden. The system was tested on 18 AD patients diagnosed with middle to late-stage AD and their caregivers aged 25 to 78 years old. Before the usage of the system, various questions regarding time and stress burden were asked from the caregivers through the Caregiver Burden Inventory (CBI) system. The system was tested for 12 weeks day and night. After the test period, the caregivers were again asked questions in the form of a user satisfaction questionnaire and CBI. Results showed that the robotic dog was a great help in managing behaviour, sadness and agitations of the AD patients. This resulted in lessening the physical, emotional, time and social burden of the caregivers to a significant extent. The system also helped in reducing the frequency of night-time wanderings and agitated mood of AD patients. The system was rated in terms of usability (89%), readability (74%), convenience (78%) and accuracy (63%), according to the survey.

Stoekle and Freund [57] also developed a smart personalized music device for the therapy of AD patients. The prototype was developed using open-source code of Java script and Hypertext Markup Language (HTML) on an Apple iPad 4. It consisted of a simple user interface with few textual features and more visual features. The 40 songs in the music player consisted of classical (10 songs), North American folk (5 songs) and 25 popular songs. The cross-sectional study took place in a care setting for memory loss and the disabled population. The study included the presence of the researcher, caregiver and the 5 selected sample of elderly patients, aged 62–69 years. The patients although not diagnosed with AD were considered a close proxy with AD patients. The data were collected through videos and direct observation. Results from the observational study show that the device can independently be used by the patients, and they enjoyed listening to the songs, and their mood was alleviated. The device was also able to automatically detect user preferences of songs.

Table 3 below provides a summary of all the studies implementing the self-contained device solutions for the diagnosis, assistance and therapy of Alzheimer’s disease.

## 4. Discussion

With the ever-increasing population of the elderly, the number of Alzheimer patients is also likely to increase. According to a 2019 report, ‘Projections of older people with dementia care in the United Kingdom, 2019–2040′, Wittenberg et al. state that currently 7.2% of people in the United Kingdom (UK) are suffering from dementia. This number is expected to increase exponentially in the years to come. Moreover, the increase in the prevalence rate of dementia is estimated to be 80% by the year 2040 [58]. This indicates a substantial increase within the next two decades, while in the United States of America (USA), it is estimated that the current number of people that are suffering from Alzheimer’s over the age of 65 years is 5.5 million [59]. According to the ‘Centre for Disease Control and Prevention, the problem of Alzheimer’s disease and related dementia is to double by the year 2060 in the USA [60]. These numbers tend to increase in other parts of the world as well. As the average life expectancy increases all over the globe, the prevalence of Alzheimer’s disease patients will tend to increase too. Global statistics state that in 2018, the total number of people with dementia was 50 million. This number is projected to rise to 152 million, which is a 204% percentage increase by the year 2050 [61].

The high incidence numbers of this vulnerable population are associated with a high cost of care. The complex physiological changes, functional assistance and neuropsychiatric needs of Alzheimer’s patients pose social and financial pressures on society, families and the overall healthcare systems. According to the research study of 2013, by Hurd et al. [62], Alzheimer’s and related dementia is the costliest disease in western countries. They state that out of all the types of dementia, the most expensive is Alzheimer’s disease. Almost 160 billion dollars were spent annually on these diseases [14,63,64]. According to the Alzheimer’s statistics report, these costs in 2018 have risen to 277 billion dollars [64]. These costs will keep on increasing with the ever-growing disease’s prevalence, as stated above. Such high costs indicate that automated, sustainable and low-cost technological solutions need to be put into practice. The intelligent solutions as identified by the review can help individuals with their everyday tasks yet provide remote up-to-date health status information to the clinicians and caregivers for timely interventions whenever required.

The main objective of this review paper was to see the advancements in recent literature and the proposed solutions that exist for the management of Alzheimer’s disease. These have the potential to assist the clinicians and Alzheimer’s patients, in the selection of appropriate solutions mapping them into their daily practice for Alzheimer’s disease management. The advantage of implementing such systems and devices in the daily life of Alzheimer patients is manifold. Moreover, the current study aimed to identify research gaps and challenges faced to orient the researchers for a better user-centric design for the intended purpose. The research studies reviewed and presented in the results section in this article have been classified according to the sensing technology (Research question 1). The details of how these technologies are playing their role in the care and management of Alzheimer’s disease have also been discussed in the results section (Research question 2). The discussion section presents an overview of these results presented in Section 3 and the associated challenges and limitations, in accordance with the focused research question of this review paper.

The breadth and depth of the reviewed research articles in the current study indicate prospective outcomes of these intelligent sensing technologies for viable solutions for Alzheimer’s disease. The range of solutions and the type of assistance they provide indicates that these intelligent sensing technologies, for the use of intricate diseases such as Alzheimer’s, are still a developing area. However, the widespread use of smart devices and miniature sensing technologies is paving the way for them to be integrated into daily living without being noticed by the users. Moreover, their ability to provide context-aware adaptive responses increases their prospects in being suitable for use in managing Alzheimer’s disease.

These solutions include both the distributed systems as well as the handheld devices and the robots. The distributed systems increase the possibilities of remote health monitoring of AD patients. These systems are suitable for in-home and care-home settings. The idea of intelligent assistive technology is to provide automated context-aware updates that can not only help in the early diagnosis but also monitor the progression of the disease. Moreover, it enables timely therapeutic interventions based on the progression speed of the disease. This helps in less damage as compared to no monitoring or even self-paced decision making as is evident by the study by Lazarou et al. [42].

### 4.1. Practical Implications of Using Intelligent Systems and Devices

The following subsection discusses the strengths of these systems with a focus on the sensors used, signals being monitored through the sensors, and how these can play a role in the early diagnosis, daily monitoring and therapy of Alzheimer’s disease patients in the comfort of their homes as well as in care home settings.

#### 4.1.1. Diagnosis of Alzheimer’s Disease

It is observed that in the case of the distributed systems, the diagnosis of Alzheimer’s disease is being done by monitoring the activities and mobility patterns of the subjects of interest. The signals monitored in the studies include walking speed [27,28], changes in mobility, absence from home, visitors, sleep patterns and toilet visits at night-time [31]. Gait speed was measured using passive infrared sensors installed in the homes of the patients. While absence from home, the presence of visitors and night-time toilet visits were monitored by magnetic door binary switches. In [29,31], RFID tags on active IoT devices carried by the patients such as mobile phones were also used to track the location and motion sensors were used to assess the environment of the user. Making using of Machine Learning algorithms, Bayesian inference and regression models, the studies use these sensor data to correlate these signals with the prevalence of cognitive decline and depression. A decline in cognition and mood changes both are symptoms of Alzheimer’s disease.

In the case of handheld devices, the most common signal monitored is the pattern of finger tapping and measuring response time, reaction time and rhythm [45,48,49]. These have been monitored by smartphones and simple home furniture which was made intelligent using magnetic switches and programming an Arduino board. The smart cupboard assessed the decline in memory which is also a common symptom of early-stage Alzheimer’s. Whereas, smart devices such as mobile phones and tablets are equipped with built-in motion and magnetic sensors. According to the study results, the response and reaction time are relatively much slower for those who suffer cognitive problems as compared to healthy controls. Mobile applications that access these sensors and calculate the response time can be of use and easily put into practice in daily lives especially in care-home setting and personal homes. The major advantage of such devices is that the results are quick, and their ubiquitous nature nowadays makes it a lucrative prospect for the early detection of Alzheimer’s disease. Moreover, simple low-cost systems, as described in [45,50], that assess memory and visio-motor coordination of individuals can easily be implemented not only aiding in a quick diagnosis, but also these kinds of systems can help in therapy purposes as well. The repetition of such tasks strengthens their motor and cognitive abilities thus aiding in slowing down the progression of the neuronal and functional damage caused by Alzheimer’s disease.

#### 4.1.2. Monitoring and Assistance of Alzheimer’s Disease Patients

The smart-home sensors, such as magnetic switches attached to doors and cupboards, pressure mats for monitoring the presence of a person in and out of the bed or chair and tags attached to the everyday household objects can give valuable activity and contextual information. This data collected by various ambient home sensors combined with some wearables that monitor the physiological parameters such as heart rate and temperature can be used to assess the erratic mood changes of Alzheimer’s patients [36]. The monitoring of the mobility and daily activities of the patients can help in assessing the autonomy, abnormal behaviours and functional decline [37,39,41] of Alzheimer’s patients. The monitoring of daily activities can also aid in assisting caregivers, alerting them during abnormal activities or risky situations such as falls and night-time wanderings [40]. The provision of assistance in daily tasks, such as cleaning, cooking etc., such as that in [38] through automated recorded messages can enhance the confidence of the elderly patients, ensuring autonomy and self-reliance. Moreover, the system introduced in [40] that assists the patient back to bed through the activation of actuators can help reduce the caregiver burden, allowing the caregiver and family members also take adequate rest.

In the case of self-contained devices, the assistance is provided by sending reminder alerts for tasks and medicine [53] or using robots that are equipped with various sensors such as cameras, temperature sensors, distance sensors, motion sensors, sensing the environment and using Natural Language Processing to interact with the patients [52]. There are commercial robots present for the aid of Alzheimer’s patients; one such example is PARO (a seal robot). The robot assesses the mood of the patient and provides her company by responding to the gestures of the patients [65]. These socially assistive robots prove to be successful mood boosters for Alzheimer’s patients.

#### 4.1.3. Therapy of Alzheimer’s Patients

The system described in [42], makes use of ambient smart home sensors, such as motion sensors and tags attached to simple everyday household objects as well as wearables to monitor the sleep and mobility patterns of the subjects. The tags were also attached to drug boxes to monitor the subject’s adherence to medicine. The study results indicated that the group which received automated reminders for medicine and regular updates from the clinicians based on their daily activities showed marked improvement in cognition as compared to the groups receiving the self-assessed, on-demand intervention and no intervention at all. This study indicates that continuous monitoring at home and sharing of the sensor data with clinicians can result in timely interventions and avoid drastic neuronal damage. Hence, such systems can practically play a vital role in slowing the progression of Alzheimer’s disease.

In the case of handheld smart devices, [55] introduced a robotic dog that could monitor mood changes, temperature and heart rate using various sensors. The results of the study indicate that the mood of the patients improves due to the interactive robot, posing less burden on the caregivers. Similar results were evident from a simple music application [57], intended to boost the mood of Alzheimer’s patients.

### 4.2. Future Research Directions for Intelligent Systems

The literature indicates the potential of intelligent systems; however, the translation of these systems to clinical practice is hampered by a few limitations. Below are listed a few shortcomings and suggestions for future research to make intelligent systems more effective for the diagnosis, monitoring and therapy for Alzheimer’s disease.

*(a)*  *Maintaining long-term Patient Databases for Distributed Systems*

A positive aspect of the distributed systems is that they can continuously or regularly share updates with the clinician. However, to make such solutions effective, database management systems are required as an integral part of the system. These systems should maintain the patient’s history to easily identify any irregular changes in the times to come. The studies that focused on the diagnosis of cognitive decline either have used datasets from the patient databases or have resulted as part of larger projects such as the dem@care and Oregon Center for Aging and Technology (ORCATECH) [27,28]. Moreover, Alberdi et al. [31] have studied 29 patients over a period of 2 years while the study in [41] resulted from the patient data of 480 homes collected over a period of 8 years. Hence, these studies indicate that the diagnosis and indication of cognitive decline is a slow process that requires time, and it may vary from patient to patient.

*(b)*  *Multi-person settings in Distributed System Designs*

Most of the studies just mentioned for database management and implementing distributed systems have taken place in societies that had smart homes built for individual patients. Although this may be a lucrative solution for people living independently, it is not suitable for homes where more than one person resides. Such examples include the family of the patient living with her or multi-person settings such as care homes. As for those homes with more than one person, the first challenge comes from recognizing the person (person identification). As the simple motion sensors used in most of the studies can only detect motions and not identify by whom the motion was made. Hence, the motion sensors placed in common areas may detect motion through anyone present, thus making the activity profiling of the individual challenging unless indoor tracking is also done.

*(c)*  *Maintaining Privacy of the Individuals*

The prospect of continuous monitoring of Alzheimer patients by using simple smart home sensors that are commercially and readily available in the market seems much advantageous. These sensors are being used for the continuous monitoring of the individuals, recognizing the daily activity patterns and then separating these from the changing or risky profiles such as less socializing, less movement and increased visits to the toilets. Moreover, these systems can monitor behavioural changes which is also an important neuropsychiatric symptom of Alzheimer’s disease. This symptom cannot be judged or indicated in short clinical visits. However, these distributed systems use cameras such as in [36,37,38] along with other sensors for the intended purpose. The drawback of the usage of cameras is the infringement of privacy.

The solutions need to be designed such that the privacy and citizenship of the patients are guarded. Although cameras can provide a more direct context-aware sensing system these also cause severe privacy infringements. Privacy is not only an issue in the sensing technology; in the case of data storage systems, there is also a potential threat of misuse of the patient data. Huge data storage systems and well maintained electronic medical records, on one hand, are lucrative aspects for the researchers but, on the other, are vulnerable to privacy risks. Hence, there is a need for clear policies for maintaining the data privacy rights of these storage systems. Policymakers and concerned organizations should come together to look into this issue.

*(d)*  *Need of Increase in Systems for Detection of Anomaly in Behaviour*

A study in [66] was conducted by Gary and Mary West Health Institute in collaboration with the Centre to Advance Palliative Care (CAPC) to understand the most challenging behaviours of Alzheimer’ patients. They surveyed over 500 caregivers by forming four in-person focus groups. According to their study, the most challenging behaviours of Alzheimer’s patients is agitation, aggression and changing moods of the patients. This problem amounted to the maximum, contributing 25% among other challenges. This challenge of abnormal behaviour was followed by other problems such as speech decline or repeated speech (12%), wanderings of the patients (10%), inconsistence (10%), late-day confusion (8%), sleeplessness and adherence to medicine (6% and 4%, respectively). Given these statistics of the problems, only behavioural issues account for one-fourth of the remaining problems. Hence, feasible and practical solutions to mitigate this issue should be researched. According to the reviewed studies, only a couple of the studies are detecting mood issues [36,55] while only one study focuses on emotional encouragement through music [57]. Hence, further research should be carried out giving more attention to incorporate behavioural changes of Alzheimer’s patients.

*(e)*  *Improving Provision of Everyday Assistance*

Intelligent sensing technologies are also being used to assist Alzheimer patients by helping them in their everyday tasks. This assistance is being provided by first observing the patients in a context that is, understanding the surroundings of the patient and then guiding them through alerts and messages. Alzheimer’s disease patients have difficulty performing their daily tasks especially in adhering to medicine due to forgetfulness along with dressing difficulties because of disorientation and confusion. Moreover, a very typical symptom and challenge experienced by caregivers is the night-time wanderings of Alzheimer’s patients. Studies have developed systems to aid these problems through soothing music, automated voice messages and video assistants [37,39,40,52]. However, these solutions also have a few limitations.

(i) *Privacy Concern:* The first is that some of the studies are using cameras which raise the privacy concerns of the stakeholders.
(ii) *Minimum User-Centric Approach:* It has been observed in the studies themselves, that AD patients feel stressed while responding to automated voice commands. Moreover, in [39], although the system alerts the caregivers of the risky behaviour of the AD patient, the acceptability and usability rate were 50% and 30% which is quite low. The application used in [53] as a reminder of medicine also deals with only 3 AD patients of which one left the study due to agitation experienced by the reminders generated by the application.
(iii) *Low Accuracy:* The study that assisted the AD patient back to bed during night-time wandering involves only one patient, and the duration of the study was not enough to reach viable conclusions. Hence, a limited number of AD patients in the studies and low accuracy rates are of concern. Moreover, Fardoun et al. [51] developed the device to aid in-person recognition for the AD patient; however, the screen size of the watch made it difficult for them to read or see the information. Hence, efforts should be made to take these considerations into account to improve the design solutions for assistance.

*(f)*  *Increase in Therapeutic Solutions*

As observed in the reviewed literature, the distributed systems and devices are also being implemented for non-pharmacological therapeutic purposes. The handheld devices either use a simple apparatus, mobile applications or virtual assistants that guide the patients in various ways through their therapies. Studies such as [50,53] aid the patient through automatic exercises for visio-motor coordination therapy, cognitive exercises or alerts aiding in memory. The smart robotic dog and the music player [55,57] also provide emotional support through music therapy. However, in the case of distributed systems [42], the only therapy that is being provided is medication alerts and timely interventions by the clinician or caregiver. This limitation indicates an inadequate number of solutions for the therapy of Alzheimer’s patients.

*(g)*  *Robust Diagnostic using Handheld Devices*

In the case of handheld devices, the studies [48,49] try to assess the cognition decline through finger tapings using a mobile screen and a simple play apparatus attached to the mobile. The diagnosis of other symptoms of functional decline related to the disease progression has been done only using self-contained devices. These diagnostic symptoms include the decline of memory [45], and visio-motor coordination [50]. These methods are quick, and the results confirm their accuracy. Although these devices have their strengths in a quick decision, a limitation mentioned by [50] is that the user can learn the patterns of visio-motor games if played again and again. This limitation can be addressed if the cognitive exercises are designed based on randomized patterns. This will make the exercise a little challenging yet useful.

*(h)*  *Involvement of Clinicians in Solution Designs*

Another important observation that was seen is that clinicians were minimally involved in the development and design of the solutions for Alzheimer’s disease. Most of the studies involve caregivers as stakeholders for their perspectives while designing the distributed systems, applications and handheld devices. Progression is a slow process, and to truly translate the technological solutions into long-term healthcare practice, the clinicians also need to be involved in the design and development phase of these technological solutions. Such a limitation implies that the proposed solutions cannot be confidently implemented by caregivers or clinicians for the management of Alzheimer’s disease. However, this constraint also indicates the challenge faced by the researchers proposing solutions for such an intricate disease. The direct involvement of the vulnerable population requires great care and precautions for many reasons. The prime reasons being the old age, easily agitated moods and frailty of such individuals. Furthermore, the widely varying symptoms between various individuals and the range of age of the patients also pose further challenges for user-centred designs. Hence, there is a need for the combined efforts where the researchers, clinicians, formal and informal caregivers can collaborate to come up with solutions that are effective and safe resulting in the user-centric design in true spirit.

## 5. Conclusions

The current study presents an overview of the intelligent technologies designed and developed for Alzheimer’s disease management, mentioned in the recent literature from 2015 to 2020. Alzheimer’s is an incurable, progressive neurodegenerative disease, which with time results in serious dysfunction of the individual suffering from it. Intelligent sensing technologies have the potential to provide low cost, sustainable clinical and home-based solutions as compared to the existing expensive and dedicated lab-oriented solutions. These can help in prolonging the stay of the patients at home and reducing hospitalizations. The continuous monitoring of the patients and regular updates being sent to the clinicians can result in timely therapeutic interventions. This can potentially aid in reducing the extensive healthcare costs spent every year on the management and care of Alzheimer’s disease. Moreover, with the existing challenge of the pandemic, these intelligent solutions become all the more necessary. Now that Alzheimer’s patients are forced to stay at home for their safety, interventions of smart technology can provide a viable solution. These technologies can help in avoiding visits to clinics and hospitals for regular check-ups and follow-ups, minimizing the risk of exposing the vulnerable elderly to other disease risks such as COVID-19. Automated solutions integrated into the daily lives of Alzheimer’s disease patients cannot only help in assisting the individuals but also reduce the stress and burden of the formal and informal caregivers. These solutions if effectively implemented can help improve the quality of life of the patients and their caregivers. Hence, awareness is to be increased among the societies and people who are directly involved in the care of Alzheimer’s patients. They need to be made aware that how these intelligent, context-aware, ubiquitous and low-cost technological solutions can help in prolonging the life of their loved one by making it easy, safe and independent. However, the practical implementation of these intelligent technological solutions is hampered by a few limitations and challenges as discussed in the discussion section above. Hence, this review article not only highlights the practical role of intelligent sensing technologies for Alzheimer’s patients but also suggests future improvements for further research in this area.

## Figures and Tables

**Table 1:** Research questions.

Type	Research Question
RQ1	What intelligent technological solutions exist in the literature that may help in the assistance and management (diagnosis, monitoring and therapy) of Alzheimer’s disease?
RQ2	How do the proposed intelligent technological solutions help in the assistance and management (diagnosis, monitoring and therapy) of Alzheimer’s disease?
FRQ	How are these intelligent systems and devices incorporating the user-centric design and what are the limitations in the existing designs implementations?

RQ: Research Question; FRQ: Focused Research Question.

**Table 2:** Distributed systems based solutions for Alzheimer’s disease.

Sr. No.	Study	Participants and Study Design	Evaluation Metric	Technology Used	Measurements	Key Findings	Study Limitations	User-Centred Design
***Diagnosis***								
1	Akl et al., 2015 [27]	N = 97; Age ≥ 70 yrs; Longitudinal study (3 years)	MMSE, CDR (at start and end of each year) and a control group.	Passive infrared sensors	Walking speed, daily home activity, visitor visits and absence from home.	Automatic detection of MCI with AUROC = 0.906 and precision-recall = 0.93 (best results with 24-week window) using Random Forest and State Vector Machine.	The smart home sensors were placed in individual’s home where they lived independently. Not suitable for the multi-person setting. Moreover, if a week activity was missed, the whole 24-week window must be discarded.	No
2	Akl et al., 2017 [28]	N = 97; Age ≥ 70 yrs; Longitudinal study (3 years)	MMSE, CDR (at start and end of each year)	Passive infrared sensors	Walking speed, home activity	The walking speed of the elderly does not slow down while transitioning into MCI. Linear modelling. AUROC = 0.716 and precision-recall = 0.706 (12-week window)	Not suitable for the multi-person setting. A small population transitioned to MCI, for validation a larger population required.	No
3	Kaur et al., 2019 [29]	N = 374; Age = 60–90 yrs; (Cross-sectional study)	Interviews from 100 patients out of the 374. (Usability, accuracy, convenience).	Radiofrequency Identification Tags (RFID) on the active IoT devices such as mobile phones	Medical history, MMSE, patient location tracking, memory exercise games data.	Diagnoses AD patients using Ontology Bayesian network (AUROC = 0.76 and F1 score = 0.934).	To build the ontology system, data were collected from a doctor. Moreover, the system uses GPS for the patients; hence, if there is no net connection, the performance might get affected.	Yes
4	Alberdi et al., 2018 [31]	N = 29; Age = 73–97 yrs; Longitudinal study (2 years) (N = 13 healthy, N = 10 at risk and N = 6 had cognitive difficulties)	ARM Curl, Timed Up and Go Test, RMANS, PMRQ and GDS (at the start of the study and every 6 months).	Magnetic switch sensors (doors), RFID tags on objects at home, motion sensors	Mobility, cognition and mood changes were monitored using smart-home sensors.	Through regression analysis, mobility, depression and cognition can reliably be detected by in-home sensors and can predict the onset of AD. Results suggest that mobility is more related to behaviour abnormalities than to cognition. Sleep patterns and toilet visits can diagnose depression.	It is difficult to assess which data from all the sensors to use. Data storage and processing of useful features are a challenge. As indicated all features are not used, and there is class imbalance. Not suitable for the multi-person setting.	No
***Continuous Monitoring***								
5	Alvarez et al., 2018 [36]	N = 18; Age = 55–94yrs; Longitudinal study (10 weeks)	Control group versus patient experimental group.	Wearable bracelet, Zenith Camera, Microsoft Kinect v2, Binary smart sensors, wireless sensor network beacons.	Physiological signals (Heart rate, skin temperature) and motion location tracking, gait, activity tracking using Kinect Microsoft and cameras.	Using multi-sensor data, the daily motion, night patterns, and detection of abnormal behaviour, fall, and gait abnormalities are evaluated. The distinction between abnormal and normal behaviour was detected with an accuracy of 98.4% using probabilistic models.	The use of cameras indicates privacy infringement, and the study included patients who did not experience cognitive impairment.	No
6	Karakostas et al., 2020 [37]	N = 109; (Mean Age = 69.8yrs) Cross-sectional	Healthy controls = 38 MCI = 44; AD = 27	Cameras, Wearable bracelets, Static sensors (on objects) and Smartphones	Camera videos to assess IADLs such as bill payment, making tea, making a phone call and walking.	Using Analysis of variance, the study shows that as the disease progresses the autonomy of the person reduces.	Use of cameras; limited IADLs and those were not a true reflection of real-life as the tasks were designed to be simple in the clinic.	No
7	Braley et al., 2019 [38]	N = 15; Age = 59–91yrs (Cross-sectional)	CDR > 0.5, Diagnosis and statistics of mental disorders (all met criteria)	Smart home sensors, speakers, actuators, TV screens	ADLs (cooking, washing, cleaning and using the telephone) were monitored	The elderly adults with more cognition problems faced difficulty while responding to voice instructions and also showed signs of stress.	Use of Cameras, limited experimenter view as only 3 cameras used, video viewing without voice, the possibility of highly subjective outcomes	No
8	Cavallo et al., 2015 [39]	N = 14 AD patients Age ≥ 80yrs (Cross-sectional) Duration depended on carer and patient availability	MMSE Qualitative assessment through experience reporting	Inertial sensors, pressure mats, Door magnetic switch sensors, Zigbee, GSM and GPS on a portable device	Home exit-stay; bed-chair monitoring, location tracking outdoor, social behaviour	Functional monitoring and alerts in case of emergency were given, but the acceptability and usability rates according to the patients and their caregivers were 50% and 30%, respectively	Not fixed duration of the experiments, all systems not utilized by all participants, low acceptability and utility rates	Yes
9	Radziszewski et al., 2017 [40]	N = 1; Age = 78yrs (Cross-sectional) (42 days)	MoCA and Dementia rating at the start and end of the study	Ambient sensors, wristwatch, Smart objects including LED bulbs, lamps, Actigraph light paths, Z-wave controllers	Day and night-time activities	K-means algorithm to detect activity and guide the patient back to bed using voice signals.	Not suitable for the multi-person setting. The study duration was insufficient to determine nocturnal patterns, limited no. of participants.	Yes
10	Lyon et al., 2015 [41]	N = 480; Age ≥ 70yrs; (Longitudinal) 8 years	MMSE, CDR	Motion sensors, magnetic switches, screens, internet, Zigbee	Mobility and activity patterns including socializing	Regression models showed that progression in cognitive decline and mood can be evaluated within six months. Wearables used as ground truth	480 single patient homes. Activity recognition not fine-grained such as in the kitchen, activity performed is washing, making tea or doing something else.	No
***Therapy***								
11	Lazarou et al., 2019 [42]	N = 18; 12 MCI; 6 AD Longitudinal 4–12 months	Diagnostic and Statistical Manual of Mental Disorders (DSM-V) MMSE before and after study 3 control groups (4MCI 2 AD each)	Off-the-shelf IoT devices, (tags attached to drug-boxes), motion sensors, wearables, cameras and ambient sensors	Motion, Sleep, Activity patterns, physiological signals and observations using depth cameras.	The second group received intervention on a self-reported basis, and the third did not receive any alerts at all. The group that received automatic alerts and reminders showed significant improvement in cognition.	Only 6 participants received interventions. Limited but complex sensor data. Single patient per home.	Yes

Table Acronyms: AD: Alzheimer’s Disease; MCI: Mild Cognitive Decline; MMSE: Mini-Mental State Exam; CDR: Clinical Dementia Rating; MoCA: Montreal Cognitive Assessment; RBANS: Repeatable Battery for the Assessment of Neuropsychological Status (RBANS); PMRQ: Prospective and Retrospective Memory Slips; GDS: Geriatric Depression Scale; AUROC: Area Under Receiver Operating Characteristic Curve; HC: Healthy Controls; ADL: Activities of Daily Life; IADL: Instrumental Activities of Daily Life.

**Table 3:** Self-contained device-based solutions for Alzheimer’s disease.

Sr. No.	Study	Participants	Intervention Used	Evaluation Metric	Measurements	Key Findings	Study Limitations	User-Centred Design
***Diagnosis***								
1	Gonzalez et al., 2019 [45]	N = 23; Age: 18–60yrs	Smart Cupboard	Control group (young Adults) vs. Experimental group (elderly) Self-reported memory test and face-name pair test	Memory assessment Reaction time	Pearson correlation results showed a high similarity between the test results and experimental results. No correlation between memory and age was found.	The assumption that only one person uses the cupboard. No cognition problem observed among individuals Battery usage	No
2	Suzumera et al., 2018 [48]	N = 31 AD (Mean Age: 74.2 yrs) 15 MCI (Mean Age: 74.3 yrs)	Mobile application	MMSE and control group 48 HC (Mean Age: 74.6 yrs)	JustTouch application evaluated the rhythm tapping, response and reaction times of fingers on the screen.	Spearman’s correlation coefficient values show significant differences between the 3 groups in terms of contact duration, rhythm and response.	Only index finger movement evaluated. A sample size of MCI patients was the bare minimum needed for the statistical test analysis.	Yes
3	Lee et al., 2018 [49]	N = 14 MCI; Age: 20s and 70s	Play apparatus with smartphone	Control group in the 20s and 70s	Reaction time	The response rate of the elderly in their 70s was 300 ms slower than that of those in their 20s. Response time of the patients was 9.64 times slower as compared to healthy controls in their 20s while 5.53 times slower than those in their 70s.	The use of Bluetooth might hinder speed accuracy.	Yes
4	Bartoli et al., 2017 [50]	N = 20 AD; Age = 57–82 yrs HC = 20 Age: 53–90 yrs	Haptic feedback robot interface	MMSE and Rey’s Figure copy-REY-Clock Drawing test Healthy Controls	Visio-motor coordination, position tracking, stability control.	The experimental group was significantly slower than HC. The attentive metric was negatively correlated with reaction time.	Session time and dominating hand difference could have affected results. With practice, the mind learns so the second session of all participants was better.	Yes
***Assistance***								
5	Fardoun et. al, 2015 [51]	N = 41, Age: 55–72 yrs	Face recognition of relatives using a smartwatch	Customized Stored data (pictures) on cloud	Tapping the watch and taking picture of the person standing in front of the patients	The system did recognize the people with moderate accuracy, but the patient had difficulty in reading the data and taking pictures.	Small smartwatch screen. Every time picture should be taken at a 90-degree angle, hardware limited (camera watch), reading difficulty, accuracy low, connectivity issues	No
6	Rudzicz et al., 2015 [52]	N = 10 AD; Age ≥ 55 yrs	Walking and interactive robot ED	Observed by caregivers and experimenter	Robot monitored ADLs of the patients through visual recognition	The robot tried to guide them if they missed a step and also tried to converse with the patients using NLP. However, the users did not interact with the robot often enough ignoring the robot prompts over 40% of the time.	Robot teleoperated, the caregiver had to intervene, AD patient could not understand complex guidelines, could guide only through simple tasks,	No
7	McGoldrick et al., 2019 [53]	N = 3 AD patients and their caregivers; Age: 59, 71, 74 10–12 weeks study duration	Mobile Application	Observation by caregivers and Tau-U acceptance and usability assessment	Reminders set by caregivers for the days and weeks.	One AD patient left the experiment after one week of the intervention being used. Others showed improvement in the memory performance	Limited people, assessment criteria mostly subjective, feeding of reminders should be done manually Difficulty turning off reminders	No
***Therapy***								
8	Lyu et al., 2020 [55]	N = 18 AD and caregivers; 12 weeks duration	Smart robotic dog and wearable system	Caregiver Burden Inventory system and questionnaire assessment	Physiological parameters, mood evaluation, EEG signals,	The robotic dog could interact with the AD patients making their mood better, on stimulus provision music played. The system could detect pneumonia accurately. Caregiver burden reduced because of the system.	Low accuracy = 63%, qualitative assessment, many wearables, EEG ear sensor, smart watch motion sensors on legs pulse oximeter band, etc., cardiopulmonary on chest, internet required for communication.	Yes
9	Stoekle et al., 2016 [57]	N = 5; Age = 62–69yrs memory loss patients	Music Player	Observational readings (experimenter)	Direct observation of the patients using the music player	The device can independently be used, and the users were able to shortlist the songs of their liking	Dedicated hardware (iPad 4), a limited number of songs, limited sample people.	Yes

Table: Acronyms: AD: Alzheimer’s Disease; MCI: Mild Cognitive Decline; MMSE: Mini-Mental State Exam; HC: Healthy Controls.

## Data Availability

Not applicable.
